# Track Detection in Railway Sidings Based on MEMS Gyroscope Sensors

**DOI:** 10.3390/s121216228

**Published:** 2012-11-23

**Authors:** Antoni Broquetas, Adolf Comerón, Antoni Gelonch, Josep M. Fuertes, J. Antonio Castro, Damià Felip, Miguel A. López, José A. Pulido

**Affiliations:** 1 Department of Signal Theory and Communications, Universitat Politècnica de Catalunya, Campus Nord UPC, 08034 Barcelona, Spain; 2 Department of Automatic Control, Universitat Politècnica de Catalunya, Campus Nord UPC, 08034 Barcelona, Spain; E-Mails: comeron@tsc.upc.edu (A.C.); antoni@tsc.upc.edu (A.G.); josep.m.fuertes@upc.edu (J.M.F.); damia.felip@gmail.com (D.F.); malopez@tsc.upc.edu (M.A.L.); mr_japs@hotmail.com (J.A.P.); 3 SENER Ingeniería y Sistemas S.A., Provença 392, 08025 Barcelona, Spain; E-Mail: antoni.castro@sener.es

**Keywords:** track detection, MEMS gyroscope, adaptive matched filtering, railway navigation

## Abstract

The paper presents a two-step technique for real-time track detection in single-track railway sidings using low-cost MEMS gyroscopes. The objective is to reliably know the path the train has taken in a switch, diverted or main road, immediately after the train head leaves the switch. The signal delivered by the gyroscope is first processed by an adaptive low-pass filter that rejects noise and converts the temporal turn rate data in degree/second units into spatial turn rate data in degree/meter. The conversion is based on the travelled distance taken from odometer data. The filter is implemented to achieve a speed-dependent cut-off frequency to maximize the signal-to-noise ratio. Although direct comparison of the filtered turn rate signal with a predetermined threshold is possible, the paper shows that better detection performance can be achieved by processing the turn rate signal with a filter matched to the rail switch curvature parameters. Implementation aspects of the track detector have been optimized for real-time operation. The detector has been tested with both simulated data and real data acquired in railway campaigns.

## 1. Introduction

The availability of global satellite-based positioning systems provides an economically viable means to improve the safety of control in low-traffic single-track railway lines still relying on human operation, for which current standard trackside automated equipment would be too costly [1–4]. Some of these systems use solely GPS-derived information to locate the train along the track [1,2], which is insufficient to reliably detect the occupied track in close parallel-track siding sections (mainly in stations) of the line. The track occupancy detection based on on-board gyroscope yaw rate measurements and processing has been proposed in a block system patent [3] which is the basis of BLOCKSAT^®^, a railway management system developed by the authors. Railway navigation based on map matching of gyroscope signals was studied in [4]. In [5,6], the possibility of using correlation techniques for railway track detection has been proposed. In railway operations, a track detection error can have catastrophic consequences, for this reason a rigorous assessment of the detection performance is a prerequisite in designing a safe signaling system. The paper contains a novel optimized track detector based on low-cost MEMS gyroscopes and a rigorous evaluation of the detection performance achieved for the range of possible velocities in a real test case.

BLOCKSAT^®^ is a satellite-based railway management system for single-track lines with low traffic density. The system allows upgrading the operations on secondary lines with optimal cost *vs.* safety and performance ratios, avoiding the installation of wayside equipment and offering real-time train location and communications between trains and control centers. The BLOCKSAT^®^ system [7] uses data fusion of EGNOS enhanced GPS with a Doppler radar odometer measurements in a Kalman filter algorithm to provide increased location and velocity accuracy and immunity against occasional GPS signal loss (for example in tunnels).

Present MEMS-based gyroscopes offer robust inertial self-contained measurements that do not depend on external signals. Commercially available MEMS gyroscopes measure turn rates with low noise standard deviations, which can deliver reliable turn detection even at slow train maneuver speeds. Simple threshold-based turn detection has been initially considered, giving under Gaussian noise assumption small false alarm and miss probabilities: 10^−8^ to 10^−10^. However in case of trains maneuvering at very low speeds, turn-rate signals become weak and threshold-based detection is not sufficient for high reliability detection. For these reasons a more robust, near optimal detection method has been conceived. BLOCKSAT^®^ achieves track-change detection by processing MEMS gyroscope measurements with an adaptive system based on the matched filter principle, which yields very low false-alarm and miss probabilities even for very low velocities resulting in low amplitude of the gyroscope output.

The paper is organized as follows: in Section 2 the gyroscope-based turn rate signals are characterized in the turnout detection application. The main performance parameters applicable to the binary track detection are defined in Section 3. A first reference simple threshold detector is studied under the assumption of Gaussian noise, resulting in good performance from moderate train velocities but insufficient detector reliability at low maneuvering velocities (Section 4). An optimized detector based on matched filtering is then presented in Section 5; the theoretical analysis shows substantial improvement over the simple threshold detector. However the matched filter implementation is challenging because of time to space interpolation and adaptive preprocessing requirements. An efficient solution is presented in Section 6, which allows the matched-filter-based turnout optimum detection with small computation requirements. The improved track detector is characterized and tested by simulation and experiments in Section 7. The paper ends with the main conclusions derived from the presented work.

## 2. Switch Geometry and Turn Rate Signals

**Figure 1.**
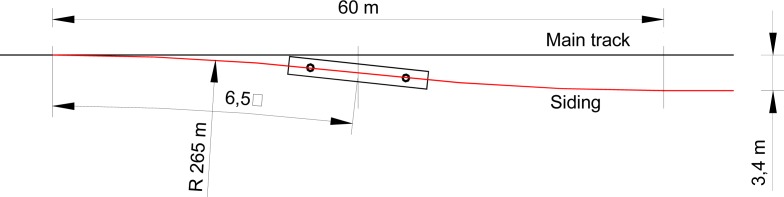
Simplified geometry of a siding switch able to divert a train from the main track in black (H0) and siding track in red (H1).

An example of simple siding geometry is shown in Figure 1, where a train can be kept on the main track H0 or diverted to a parallel siding track H1 to perform crossing operations.

**Figure 2.**
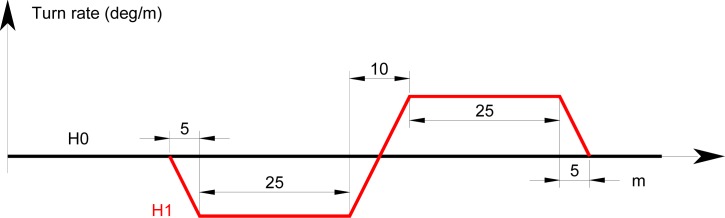
Simplified turn rate signals for main track in black (H0) and siding track in red (H1).

A MEMS gyroscope provides a voltage signal that is proportional to the yaw turn rate. In this paper the gyroscope is assumed to be integrated in a navigation system installed in the train head. Using a common external direction of reference, in a first approximation, the heading angle of a railway vehicle can be assumed to be the angle of the track tangent at the vehicle position. However the measured turn rate differs from the tangent approximation because of the large typical distances between car bogies, which cannot be neglected with respect to the turnout length. For the geometry of Figure 1 the simulation of a gyroscope signal obtained on-board of a train head with 10 m distance between bogies centers, results in linear turn rate transitions at beginning and end of the rail road curved segments. The resulting turn rate when the train is diverted to the siding track (H1) is shown in Figure 2. This simple representative turn rate signal will be used as a reference for theoretical and simulation modeling of the proposed detectors. The H0 straight track results in a null turn rate and is also shown as a reference in Figure 2.

The resulting signal is increased, with respect the track tangent, by the distance between the train head bogies; this must be taken into account when characterizing the turnout yaw rate signature for the optimized detection systems, such as the one presented in Section 5, that require an accurate knowledge of the turn rate signal.

**Figure 3.**
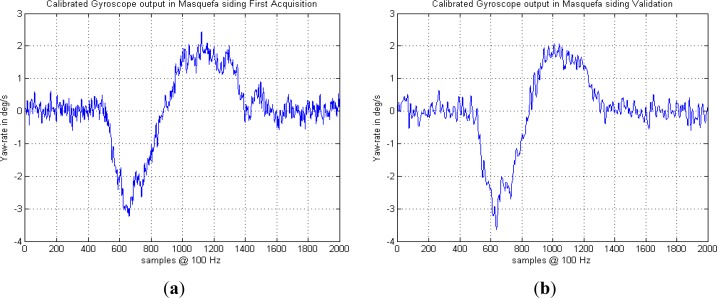
Yaw rates of a turnout in the Masquefa station measured in two experimental campaigns with different hardware and software. (**a**) December 2007. (**b**) December 2011. Small differences are due to gyroscope noise and slightly different train velocities and decelerations during the acquisition.

The experimental railway turn rate acquisitions with MEMS gyroscopes reveal the geometrical details of a real siding implementation consisting of a standard switch followed by a transition curve section to achieve a siding parallel track. The tests have shown a remarkable repeatability of the gyro turn rate measurements, which has been maintained for years, even when using different train heads, gyroscope models and acquisition equipment. As an example, Figure 3 shows an acquisition carried out in the Masquefa station turnout of the Igualada-Martorell line of Ferrocarrils de la Generalitat de Catalunya (FGC) in December 2007 compared to a second acquisition of the same turnout carried out in December 2011. Different gyroscope models, acquisition hardware and processing software, low pass filters, *etc.* where used; however the gyroscope yaw rates are very similar, including small systematic variations superimposed to the basic oscillation pattern.

## 3. The Binary Track Detection

**Figure 4.**
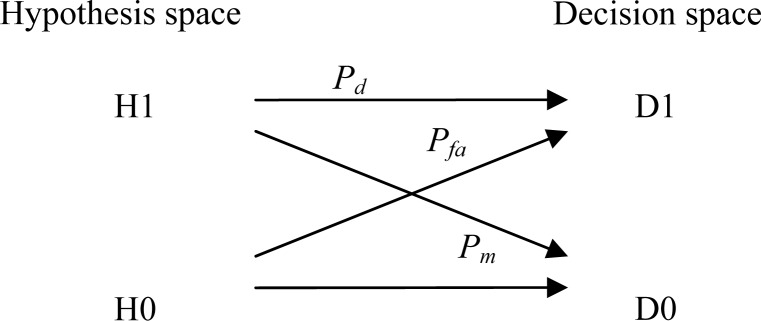
Binary hypothesis of a real situation: H1: train diverted to siding, H0: train has remained on main track. Possible detector decisions: D1: turnout detected, D0: turnout not detected.

The detection of a turnout deriving a train to a parallel track siding from the main track can be obtained by processing the turn rate signal provided by a MEMS gyroscope. The turnout detection is a binary decision, similar to a single bit detection in telecommunications or target detection in radar [8,9]. Assuming a single track line, at a certain Kilometric Point (KP) where the track splits into a main track and a siding parallel track, two hypotheses are possible: H1 means the train has been diverted to the siding track, and H0 means that the train remains on the main track. A turnout detector after processing the gyro signal will take a decision between two possible outcomes: D1 corresponds to a positive detection of the turnout, locating the train on the siding, whereas D0 means that the detector after processing the gyro, data has decided that the train head has followed the main track. For every hypothesis, correct and incorrect decisions are possible as depicted in Figure 4. The quality of the detection is usually assessed in terms of *P_d_* (Probability of detection) and *P_fa_* (Probability of false alarm). In some cases it is also useful to state the Probability of miss *P_m_* which is complementary to *P_d_* : *P_m_* = 1 − *P_d_*.

## 4. Performance of a Simple Turn Rate Threshold Detector Using a MEMS Gyroscope

**Figure 5.**
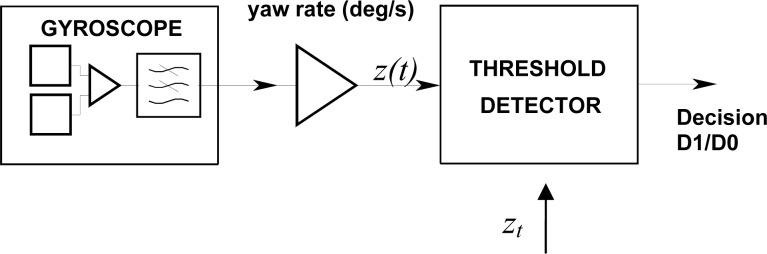
Simple threshold detector based on the comparison of a low-pass filtered turn rate *z(t)* with a preset threshold *z_t_*. D1 is decided when *z(t)* ≥ *z_t_*, D0 is decided when *z(t)* < *z_t_*.

A simple yaw rate threshold comparator was used as a first reference detector as shown in Figure 5. We characterize the performance of this detector using the ADXRS614 gyroscope [10], a low-cost MEMS sensor manufactured by Analog Devices that has been integrated in the BLOCKSAT^®^ prototype system [7]. The ADXRS614 contains two polysilicon resonators producing Coriolis force in case of rotation. The yaw rate induced Coriolis motion is sensed by a capacitive pickoff fingers structure placed orthogonally to the resonator motion. The principle of operation is described in [11,12]. The resulting signal is fed to a series of gain and demodulation stages that produce the electrical rate signal output. The dual-sensor design provides external g-forces and vibration rejection.

This gyro device has a typical yaw rate measurement range up to ±75 deg/s with a typical noise density of 0.04 deg/sec/√Hz at 25 °C. The sensor specifications [10] show a noise spectrum approximately flat within the selected noise bandwidth, which is set by an external capacitor.

The evaluation of the detection performance is based on the error characterization of the MEMS gyroscope. Neglecting the linear accelerations sensitivity and cross-axis coupling the measured yaw rate *z* can be modelled as [13]:

(1) z=(1+S)Ω+b+n

where Ω is the actual yaw rate, *S* is the scale factor of the sensor, *b* is the gyroscope bias and *n* is the gyroscope noise. Both the scale factor and bias are temperature dependent [14]; the ADXRS-614 includes a temperature sensor allowing the thermal compensation of these errors. In our case, since the rail track is accurately known, both gyroscope scale factor and bias can be frequently estimated and compensated for example at curves with constant radius of curvature (scale factor) and at straight track sections (bias). For these reasons in the proposed detection system where the gyroscope output is only used in a narrow time-window corresponding to the train head turnout transition, the dominant term of the measurement error is the yaw rate noise which can be characterized as a stochastic process with some probability distribution and autocorrelation function [15].

A typical noise histogram provided by the gyroscope manufacturer suggests a Gaussian probability density function, which is a usual assumption for MEMS gyroscopes statistics based on physical considerations and experimental data [13,16–19]. The Gaussian assumption was confirmed with a signal acquisition histogram of the used gyroscope in static condition during three hours at 100 samples/s. Given the short time span of the intended signal detection and the proposed frequent gyroscope calibration on appropriate known straight or curved segments, the sensor scale factor and bias-compensated noise process will be assumed stationary.

In addition to the probability distribution, the stochastic process autocorrelation function is needed to describe how fast the gyroscope noise evolves with time. A white noise is often used as a physical error source model [16,17] which under limited band restrictions often becomes a Gauss-Markov model of first or higher order, related to autoregressive models in the discrete domain [13,16]. According to the internal architecture of the ADXRS-614 gyroscope, the capacitive sensor is followed by an integrated first order low pass filter with an exponential impulse response whose time constant is set by an internal resistor R = 200 kΩ and an external capacitor C [10]. In this case, assuming a white noise sensor at the filter input with constant power spectral density Φ*_w_*(*f*), the resulting first-order Gauss-Markov power spectral density Φ*_g_*(*f*) at the gyroscope output is:

(2) $$\Phi_{g}\left(f\right)=\Phi_{w}\left(f\right){\left|H\left(f\right)\right|}^{2}=\frac{\sigma^{2}}{\pi B}\frac{1}{1+{\left(f/B\right)}^{2}}$$

where *H*(*f*) is the filter transfer function and *σ*^2^ is the noise variance at the gyroscope output. The time constant τ = RC sets the filter −3 dB Bandwidth *B*:

(3) $$B=\frac{1}{2\pi RC}$$

The resulting noise is a Gaussian process with an exponential autocorrelation function [13] where the filter time constant τ defines the correlation interval of the gyro noise:

(4) $$R_{g}\left(t\right)=\sigma^{2}e^{-\frac{\left|t\right|}{\tau }}$$

The noise variance can be obtained as *σ*^2^ = *N*_0_*B_N_*, where *N_0_* is the noise unilateral spectral density and *B_N_* is the sensor noise equivalent bandwidth, defined as the bandwidth of an ideal rectangular response filter that would provide the same noise power as the actual filter at its output:

(5) $$B_{N}≜\frac{\int_{0}^{\infty }{\left|H\left(f\right)\right|}^{2}df}{{\left|H\left(0\right)\right|}^{2}}=\frac{\pi }{2}B$$

In many applications *B* can be used as a good approximation to *B_N_*. In our case *B_N_* is around a 50% larger than *B*, because of the small roll-off of the first order filter response [8]. In order to estimate correctly the noise power and to be more conservative when selecting appropriate sampling rates of the processing stages, we will characterize the design bandwidth using the noise equivalent bandwidth *B_N_* instead of the −3 dB bandwidth *B*.

In many cases of interest the train can stop immediately after being diverted into a siding track. For this reason the turnout detection will be based on the gyro yaw rate short term signal observation when the train head transits on the switch section of the track. A map-matching approach, which has been proposed in railway navigation [4] has been ruled out in this case, as it requires tracking the train head evolution after the switch, which is not possible when the train stops. Moreover, the fact that the lateral track of a siding converges quickly to the main track would difficult the discrimination capability of a map-matching algorithm.

Due to the short term observation of the gyro signal, the proposed siding detection requires an accurate train location on the track in order to carry out the detection at the peak of the yaw rate. In the BLOCKSAT^®^ system, this information is supplied by the navigation subsystem based on the position and velocity data supplied by an EGNOS enhanced GPS and a Doppler radar odometer (both duplicated). The train position on the track is specified to have a maximum error of 10 m. All sensors (gyroscope, odometer, GPS receiver), and processing and communication subsystems are duplicated to conform the system reliability and availability specifications.

The binary detection theory in the case of signals in additive Gaussian noise is well known in communications, radar and sonar applications and has been applied to the present case. Following the Neymann-Pearson Criterion [20] we constrain a minimum probability of false alarm and establish a test to maximize the probability of detection. In the stationary noise case, a simple threshold detector can be used for this purpose, where the gyroscope yaw rate signal is compared with a preset yaw rate threshold level as shown in Figure 5. If the low-pass filtered turn-out rate *z*(*t*), provided by the gyroscope, equals or exceeds a preset threshold *z_t_*, D1 is decided; otherwise the decision is D0.

**Figure 6.**
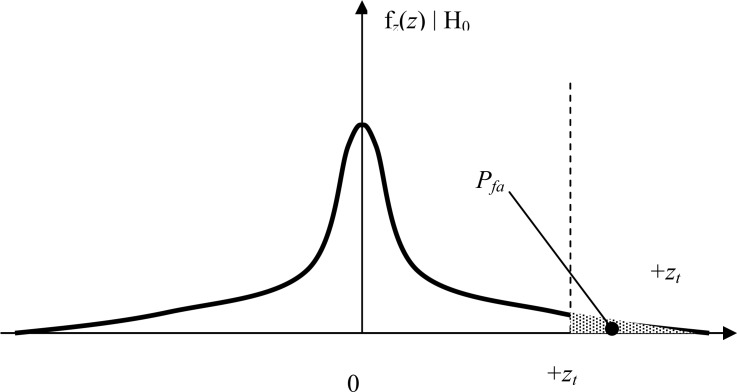
Probability of false alarm of a positive turnout detection D1|H0. The threshold value in deg/sec is *z_t_*.

In the hypothesis H0 the train head follows a straight trajectory, and assuming a well calibrated and temperature-compensated gyroscope, the probability of false alarm, at a given observation time, can be expressed as the probability that the Gaussian noise voltage equals or exceeds the threshold (see Figure 6):

(6) $$P_{fa}=\frac{1}{\sqrt{2\pi }\sigma }\int_{z_{t}}^{\infty }\text{exp}\left(-\frac{n^{2}}{2\sigma^{2}}\right)dn$$

**Figure 7.**
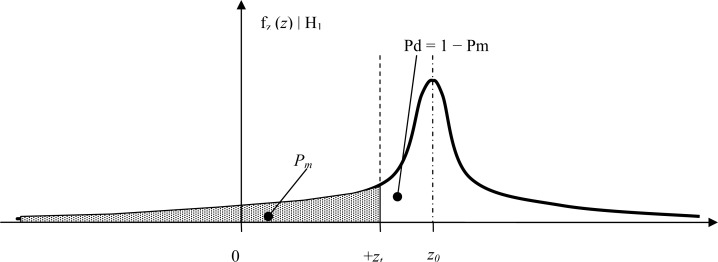
Probability of detection in a positive turnout D1|H1. The threshold value in deg/sec is *z_t_* and the turnout induced yaw rate is *z_0._*

Under the hypothesis H1 the detection probability can be calculated in a similar way (see also Figure 7):

(7) $$P_{d}=1-P_{m}=1-\frac{1}{\sqrt{2\pi }\sigma }\int_{-\infty }^{z_{t}}\text{exp}\left(-\frac{{\left(z-z_{0}\right)}^{2}}{2\sigma^{2}}\right)dx$$

To determine the detector threshold we need to estimate the gyro noise variance *σ*^2^. Since noise power is proportional to the sensor bandwidth it is convenient to reduce the gyro bandwidth to the minimum value compatible with the gyro turn signal of hypothesis H1. In the studied turnout the speed is limited to a maximum of 30 km/h; assuming a higher speed limit of 50 km/h, the gyro would eventually provide a signal that can be approximated by a sinusoidal cycle with a period *T_trn_* of around 5 s, and an associated bandwidth equal to the inverse of time duration *T_trn_*. In order to better preserve the signal original shape the −3 dB bandwidth has been set as twice the signal bandwidth:

(8) $$B=2/T_{\mathrm{trn}}=0.4\;\text{Hz}$$

In this case the resulting noise variance at the gyro output is *σ_z_* = 31.7 × 10^−3^ deg/s. From Equations (6) and (7), using the complementary error function we obtain:

(9) $$P_{fa}=\frac{1}{2}\mathrm{erfc}\left\{\frac{z_{t}}{\sigma_{z}\cdot \sqrt{2}}\right\}$$

(10) $$P_{d}=1-P_{m};\;\;\;\;\;\;\;\;\;\;\;\;P_{m}=\frac{1}{2}\mathrm{erfc}\left\{\frac{z_{0}-z_{t}}{\sigma_{z}\cdot \sqrt{2}}\right\}$$

where:

(11) $$\mathrm{erfc}\left(x\right)=\frac{2}{\sqrt{\pi }}\int_{-x}^{\infty }\text{exp}\left(-t^{2}\right)dt$$

The threshold setting *z_t_* is a trade-off between tolerable false alarms and detection misses. For safety reasons extremely low error probabilities are required in railway signaling applications, the maximum allowable value depending on the error consequences and the associated safety level specification [21]. Accordingly we adopt a stringent requirement of a probability of error equal to or below 10^−9^, to assess the feasibility of a railway turnout detector based on low-cost MEMS gyroscopes. In the analyzed detector a required *P_fa_* = 10^−9^ results in a threshold:

(12) $$P_{fa}=\frac{1}{2}\mathrm{erfc}\left\{\frac{z_{t}}{\sigma_{z}\cdot \sqrt{2}}\right\}=10^{-9}\to z_{t}≅6\sigma_{z}=0.19\;\text{deg}/\text{s}$$

The probability of detection under hypothesis H1 will be conditioned by the train velocity. For a minimum velocity *V_min_* = 5 km/h and a turnout curvature radius *R_c_* = 265 m we obtain a turn rate of *z_0_* = 0.3 deg/s, in which case the probability of a miss is:

(13) $$P_{m}=\frac{1}{2}\mathrm{erfc}\left\{\frac{z_{0}-z_{t}}{\sigma_{z}\cdot \sqrt{2}}\right\}=2.6\cdot 10^{-4}$$

The resulting miss probability is much higher than the 10^−9^ required limit. This implies that a high level of operation safety with both *P_fa_* and *P_m_* equal to or lower than 10^−9^ cannot be obtained with this simple detector at very low speeds. The peak signal-to-noise ratio is defined as the peak signal power at the instant of maximum amplitude *t_m_* divided by the average noise power (variance) of the gyroscope output:

(14) $$\hat{S}/N≜\frac{z^{2}\left(t_{m}\right)}{\sigma_{z}^{2}}$$

In the present case *z(t_m_)* = *z_0_* and *Ŝ* / *N* = 19.52 dB. The minimum *Ŝ* / *N* to achieve the required *P_m_* = 10^−9^ corresponds to *z_0_* ≅ 12 *σ_z_* and (*Ŝ* / *N*)_min_ = 21.58 dB. This minimum *Ŝ* / *N* is achieved with a train velocity of 6.3 km/h, assuming ideal operation of the gyroscope. It is clear that in order to operate with a reasonable safety margin the detector performance must be improved substantially.

## 5. Optimized Detection Based on Matched Filtering

Correlation based signal detection in low signal-to-noise ratio situations is usual in communication systems [8], radar [9] and navigation [22]. If the shape of the signal to be detected is known, a matched filter provides the maximum possible peak signal-to-noise ratio. For this reason the proposed optimized detector is based on the matched filter, which improves remarkably the invariant low-pass filter performance of the simple threshold detector described in Section 4.

In a first simplified analysis we assume that the velocity of the train is known and constant, and therefore the turnout yaw rate signal can be predicted from the track geometry. This allows to define the matched filter impulse response *h_m_(t)*, which, with additive Gaussian white noise is a time-inverted replica of the noise-free turnout yaw rate *z(t)*[8] as follows:

(15) $$h_{m}\left(t\right)=A\cdot z\left(t_{m}-t\right)$$

The deterministic component *y(t)* at the output of the matched filter is the time-shifted autocorrelation of the yaw rate signal *z(t)*, except for an additional arbitrary amplitude *A* and an arbitrary time shift *t_m_* at which the peak of the autocorrelation is obtained. In practice *A* and *t_m_* are constants that depend on the filter implementation and can be determined by calibration. Since these constants do not impact the final signal-to-noise ratio it is usual to take *A* = 1, *t_m_* = 0 to simplify the theoretical analysis. The resulting peak signal-to-noise ratio at a filter output can be obtained as the maximum signal instantaneous power *y*^2^(*t_m_*) divided by the noise variance $\sigma_{y}^{2}$; when using a matched filter the signal-to-noise ratio can be easily obtained knowing the energy *E* of the turnout yaw rate signal and the noise unilateral spectral power density *N_0_*[8]:

(16) $$\hat{S}/N=\frac{y^{2}\left(t_{m}\right)}{\sigma_{y}^{2}}=\frac{2E}{N_{0}}$$

In the case of the Masquefa station turnout, the estimated average turnout yaw rate of ±0.277 deg/s at the minimum velocity of 5 km/h (see Section 4) is a worst case for turnout detection since the yaw rate signal is very small; however the turnout time in this case is *T_tnt_* = 50.4 s, allowing the matched filter to increase the gyroscope signal-to-noise ratio due to the integration time of the signal autocorrelation. The noise unilateral power spectral density is obtained from the gyroscope specifications, resulting in the peak signal-to-noise ratio:

(17) $$\hat{S}/N=\frac{2E}{N_{0}}=\frac{2\int_{-\infty }^{\infty }z^{2}\left(t\right)dt}{{\left(0.04\;\text{deg}/s\right)}^{2}/Hz}=4860=36.87\;dB$$

The matched filter provides a substantial increase of the peak signal-to-noise ratio (+16 dB) with respect to the simple detector. Due to the large margin achieved in signal-to-noise ratio there is a wide range of possible thresholds that would satisfy the requested probability of decision error, *P_fa_* and *P_m_* ≤ 10^−9^. Assuming the detection can be performed at the peak of matched filter response we can select the threshold *y_t_* that minimizes the probability of a decision error using Equation (9) and:

(18) $$P_{d}=1-P_{m};\;\;\;\;\;\;\;\;\;\;\;\;\;\;\;P_{m}=\frac{1}{2}\mathrm{erfc}\left\{\frac{\hat{y}-y_{t}}{\sigma_{y}\cdot \sqrt{2}}\right\}$$

From Equations (9), (17), (18) and Figures 6 and 7 it is easy to see that selecting the threshold as the half peak turn rate value *y_t_* = *y*(*t_m_*)/2 results in *P_fa_* = *P_m_* ≪ 10^−9^. A remarkable safety margin is obtained even at the minimum manoeuvring velocity considered, which means the expected detection performance is robust in front of non-ideal subsystems performance like sensor degradation or matched-filter impulse response errors. At higher train velocities the yaw rate levels will increase proportionally, whereas the integration time provided by the matched filter will decrease. In any case the signal-to-noise ratio will always increase with higher train velocities from the baseline calculation of Equation (17), since the energy is proportional to the square of the yaw rate amplitude (∝ velocity^2^) and proportional to the integration time (∝ 1/velocity).

**Figure 8.**
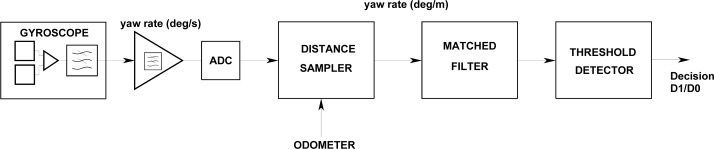
Gyroscope processing for robust track occupancy detection.

The Equations (16) and (17) provide the maximum S/N values that can be obtained by an ideal system. The practical implementation of the matched filter in the present case is complicated due to the velocity-induced amplitude and time scale factors affecting the yaw rate signal. To reach the optimum S/N, the minimum sampling rate of the gyro signal is constrained by the noise equivalent bandwidth, resulting in a variable length correlator structure with a very high number of coefficients at low circulation velocities. For these reasons a two-stage design of the matched filter based on velocity controlled multi-rate processing is proposed in order to get close to optimum matched filter operation with moderate computation resources. The processing chain is shown in Figure 8, where an invariant matched filter is preceded by an adaptive low-pass filter and a distance sampler.

The yaw rate signal delivered by the gyroscope is first amplified in order to adapt the expected output levels to the dynamic range of the 10 bit Analogue to Digital Converter (ADC). The amplifier response is low-pass with a frequency cut of 200 Hz in order to increase the rejection of a 14 kHz spurious signal which is the gyroscope mechanical resonance frequency [10]. In order to implement a train speed adaptive processing the gyroscope signal is digitized with a sampling frequency *f_S_* = 100 Hz. At this sampling frequency the gyroscope output bandwidth is not critical. Selecting an external capacitor of 50 nF in the ADXRS614 gyroscope circuit [10], its output noise equivalent bandwidth is limited to *B_N_* = 25 Hz.

**Figure 9.**
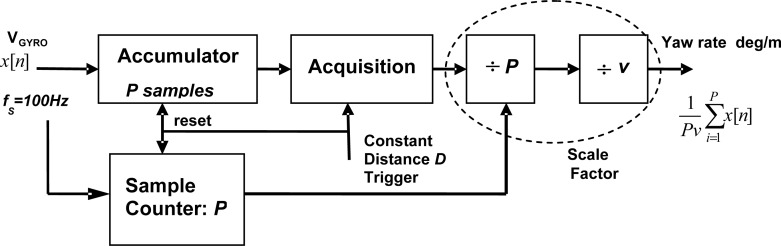
Distance sampler accumulator including train velocity scaling factors.

Using the train odometer as travelled distance sensor, the yaw rate samples are processed by an adaptive accumulator (Figure 9) that integrates the gyroscope measurements obtained during constant distance intervals. This integration allows two basic functions: adaptive yaw rate low-pass filtering and gyroscope signal scaling to a spatial turn rate.

For example, if the train velocity is reduced with respect to a reference value, the train head yaw rate in a curve decreases while the time scale of the signal is enlarged both in the same factor. In addition if we wish to process the yaw rate signal with a matched filter it would be convenient to spatially scale the signal in degrees/m in order to get an invariant geometric pattern with respect to the train velocity. This scaling is performed by the constant distance accumulator followed by an adaptive amplitude correction. The amplitude correction factor takes into account the number of accumulated *P* samples during the integration distance and a velocity-dependent scale factor relating the time and space yaw rates as shown in Figure 9.

The combination of the scaling factors 1/*P* and 1/*v* of Figure 9 is approximately constant except for quantization errors due to integer number of samples. If the integration distance is *D* and the train velocity is *v*, the number of integrated samples *P* is:

(19) $$P≅\frac{D/v}{T_{S}}\;\;\;\;\;\;\;\text{if}\;\;\;\;\;\;\;\;\;P\gg 1$$

where *T_S_* is the sampling period of the A/D converter. Note that *P* is proportional to the integration distance *D* and the A/D sampling frequency *f_S_*, and inversely proportional to the train velocity. In the test railway case *D* = 2 m and *f_S_* = 100 Hz, resulting in a large number *P* even for the fastest expected train velocities (50 km/h). Since the constant distance D trigger is not synchronized with the A/D sampling frequency, the integer number P will exhibit a +1 count jitter along the accumulator operation approaching in average the fractional value of the right side of Equation (19). Under the approximation of Equation (19) the scale factor to normalize the accumulator reading to obtain a spatial turn rate in degrees/meter is approximately constant:

(20) $$\frac{1}{P}\cdot \frac{1}{v}≅\frac{1}{f_{S}\cdot D}\;\;\;\;\text{s}/\text{m}$$

**Figure 10.**
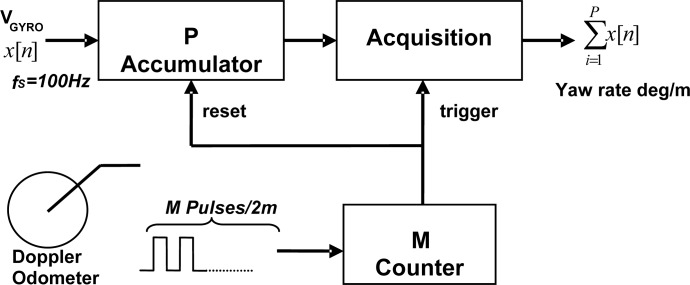
Simplified Distance sampler accumulator.

For this reason the adaptive 1/P and 1/v factors have been dropped in the final implementation of the distance sampler shown in Figure 10 using part of the available FPGA gates of the BLOCKSAT^®^ on-board navigation system. The spatial step of 2 m adopted for temporal-to-spatial yaw rate interpolation delivered by the accumulator provides a sufficient number of samples for the matched filter processing even for short turnouts. A Doppler radar-based odometer delivering digital measurements of velocity, traveled distance and pulse generation has been used avoiding wheel slippage errors.

**Figure 11.**
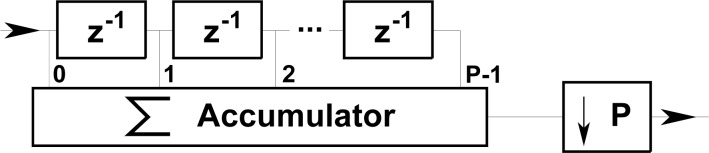
The accumulator modeled as an adaptive P-order moving average low-pass filter, followed by a decimator.

To analyze the adaptive low-pass filtering function of the accumulator, it may be considered as equivalent to a Finite Impulse Response (FIR) filter with a variable order P dependent on the train velocity as depicted in Figure 11.

The power frequency response of the accumulator is:

(21) $${\left|H\left(f\right)\right|}^{2}=\frac{{\text{sin}}^{2}\left(\pi fP/f_{S}\right)}{{\text{sin}}^{2}\left(\pi f/f_{S}\right)}$$

The resulting noise-equivalent bandwidth is *B_N_* = *f_S_*/(2*P*) where *f_S_* is the sampling frequency of the ADC and *P* is the number of accumulated samples, inversely proportional to the train velocity (Equation (19)). Therefore at low speeds the accumulator integrates a higher number P of samples, which on one hand compensates for the low yaw rate amplitude and, on the other, reduces the noise equivalent bandwidth and the resulting noise power at the output. After decimation the sampling rate is reduced to *f_s_*/*P*, which corresponds to the intended constant spatial sampling every 2 m. Selecting a high ADC sampling frequency *f_S_* reduces the discretization and jitter error resulting from the integer number of integrated samples *P* in the accumulator operation. The worst case corresponds to the highest expected train velocity which is limited to 50 km/h in the switch sidings, resulting in *P* = 14 (+1 jitter) and a maximum jitter error of 7.14% which will be averaged in the matched filter with little impact on the peak correlation output.

## 6. The Track Detector Performance

Since the signal provided by the distance sampler is not affected by the vehicle velocity or accelerations, a simple spatial matched filter can be applied to the integrated gyro signal to obtain the optimal peak signal-to-noise ratio. The track decision is obtained by comparing the signal sample at the peak position of the matched filter output with an appropriate turn rate threshold. The resulting probabilities of detection and false alarm can be calculated with the approach described in Section 4.

With the proposed spatial sampling, the matched filter length is constant and sufficiently small for real-time operation. In the studied case the matched filter impulse response has a length of 35 samples corresponding to the 70 m long H1 turn rate signal of Figure 2. Instead of reproducing faithfully the train yaw rate waveform, the filter can be simplified by using only +1 and −1 constant coefficient weights. In this case only additive operations are carried out on a shift register fed with the yaw rate measured samples; this allows a recursive implementation of the FIR filter with very small computational load [23]. The constant amplitude approximation of the filter impulse response results in a signal-to-noise ratio degradation with respect to the ideal design case characterized by Equations (15) and (16). For a non-perfectly matched filter both the peak power of the signal at the time of maximum amplitude *t_m_* and the average noise power must be calculated to obtain the suboptimal signal-to-noise ratio:

(22) $$\hat{S}/N=\frac{{\left|y\left(t_{m}\right)\right|}^{2}}{\sigma_{y}^{2}}=\frac{{\left|z\left(t\right)*h\left(t\right)\right|}^{2}{}_{t=t_{m}}}{\int_{0}^{\infty }N_{0}{\left|H\left(f\right)\right|}^{2}df}$$

where *h*(*t*) is the impulse response of the actual filter, *H*(*f*) its Fourier transform, and * denotes the convolution operator. When the train approaches a siding, the matched filter impulse response, obtained from a reduced set of geometric coefficients available in the navigation Rail Track Data Base (RTD), is dynamically loaded in the BLOCKSAT^®^ system Single Board Computer. The track detector operates locally, by real-time processing the gyro data, acquired during the train head passage through the switch. The spatial window of acquisition is dimensioned according to the switch length, the maximum position error, specified as 10 m, and an additional safety margin of 20 m.

The performance of the proposed processing chain has been simulated on MATLAB^®^-Simulink at 5 km/h and 50 km/h, which are the minimum and maximum nominal velocities of operation in siding areas.

**Figure 12.**
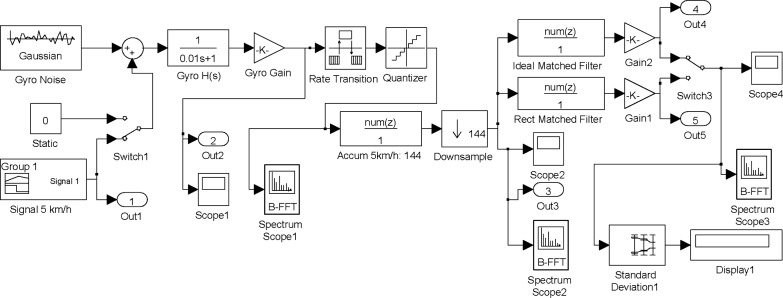
MATLAB^®^-Simulink model of gyroscope and proposed 2-stage optimal processing. The simulation parameters shown in the figure correspond to a 5 km/h test.

**Figure 13.**
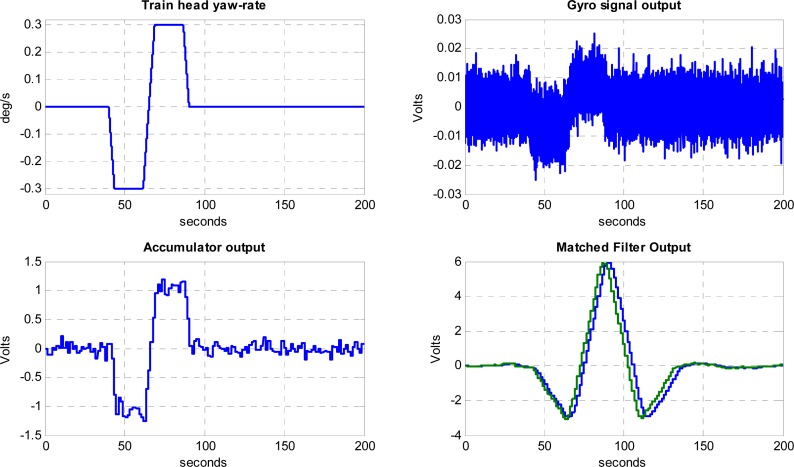
MATLAB^®^-Simulink results of a 5 km/h simulation. Five signals are shown corresponding to the output ports 1 to 5 of the Simulink model. Top-left: port 1: actual yaw rate. Top-right: port 2: gyro output. Bottom-left: port 3: Accumulator output. Bottom-right: ports 4, 5: Blue is the ideal Matched Filter Output and Green is the Rectangular approximated Matched Filter.

**Figure 14.**
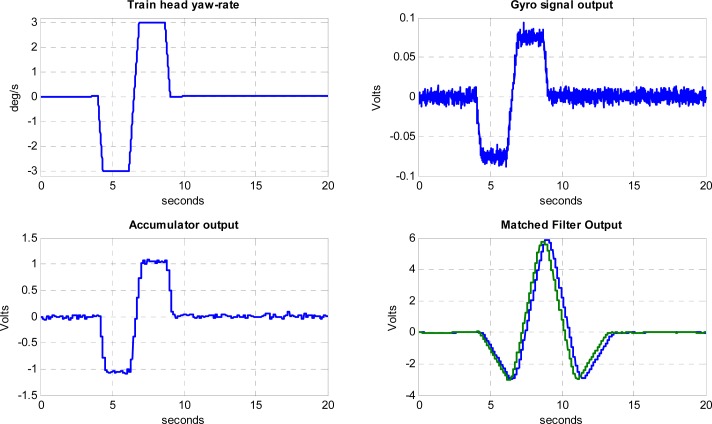
MATLAB^®^-Simulink results of a 50 km/h simulation. Five signals are shown corresponding to the output ports 1 to 5 of the Simulink model. Top-left: port 1: actual yaw rate. Top-right: port 2: gyro output. Bottom-left: port 3: Accumulator output. Bottom-right: ports 4, 5: Blue is the ideal Matched Filter output and Green is the rectangular approximated Matched Filter.

Figure 12 shows the Simulink model on which the performance of the proposed processing chain has been simulated. Simulations results at 5 km/h and 50 km/h, which are the minimum and maximum nominal velocities of operation in siding areas, are presented in Figures 13 and 14. The first blocks at the left side of Figure 12 correspond to internal gyro Gaussian white process with the variance resulting from the noise spectral density specifications of the gyro. An arbitrary-signal generator block reproduces the actual yaw rate evolution of a train head following the track shown in Figure 1, resulting in the trapezoidal signal already presented in Figure 2. The yaw rate evolution with time is available at the port Out1 of the simulator and represented in Figure 13 Top-Left.

The horizontal axis of Figure 13 plots represents the transit time in seconds on the turnout section of the rail road. A first-order low-pass filter reproduces the output circuitry of the gyroscope sensor, where a noise equivalent bandwidth *B_N_* = 25 Hz has been selected in order to reduce the impact of aliasing on the total noise power when sampling the gyro signal at 100 Hz. After the low-pass filter a gain block has been included in the simulated chain in order to model the gyro yaw rate-to-voltage sensitivity. The port Out 2 shows the simulated electrical signal provided by the gyro, which is represented in Figure 13 Top-Right. It can be seen that at low velocities the signal-to-noise ratio is very low. The gyroscope signal is sampled with a rate transition block modeling the ADC sample & hold and quantized with a 10-bit resolution; in this way the quantization noise induced by the ADC converter is included in the model.

A FFT analysis of the digitized noise-only signal shows a noise power spectrum very close to the theoretical values. Since no odometer has been included in the simulator, the accumulator performance has been assessed using a FIR filter and decimation equivalent model shown in Figure 11.

**Figure 15.**
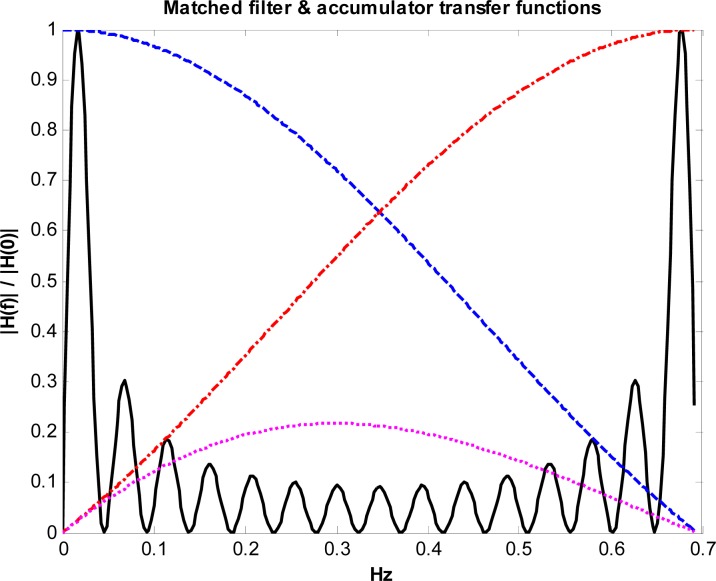
Absolute values of the normalized frequency transfer functions of the matched filter (constant coefficient version) and of the accumulator obtained at 5 km/h velocity. The black solid line corresponds to the matched filter; the blue dashed line is the accumulator fundamental response. The first accumulator aliases resulting from decimation are represented in red dot-dashed curve and magenta dot curve.

In the 5 km/h simulation, approximately 144 samples of the gyro signal are accumulated and delivered to the matched filter every 2 m. This results in a drastic sampling-rate reduction from *f_S_* = 100 Hz to *f’_S_* = 0.694 Hz. At this reduced sampling rate the noise spectral density at the output of the FIR filter modeled by Equation (20) extends beyond the alias-free region, which might cause a degradation of the signal-to-noise ratio due to aliasing. Fortunately the matched filter has as a very narrow band-pass frequency response very close to the origin, as shown in Figure 15, where the impact of aliasing is very small due to the periodic nulls of the FIR filter response in Equation (21). Moreover, the null at the origin of the matched filter transfer function implies the output will not be affected by possible gyroscope bias. At the reduced accumulator sampling-rate the noise spectrum density is white, since the samples are completely uncorrelated; this flat noise spectral density justifies the hypothesis of design of the matched filter in Equation (15).

The Bottom-Left plot on Figure 13 shows the signal available at the accumulator output. The bandwidth reduction of the accumulator increases notably the signal-to-noise ratio of the yaw rate signal, which is further improved by the narrow band response of the matched filter. Two versions of the matched filter have been simulated, namely an ideal-impulse-one derived from the expected incoming yaw rate signal, and a simplified constant-weight rectangular version that can be recursively implemented. Both matched-filter outputs (Figure 13 Bottom-Right) are very similar, with slight signal loss in the case of the constant-weight filter coefficients. The peak response of the filters occurs when the moving yaw rate signal is aligned with the matched filter spatial waveform; this means that, neglecting the processing delay, the ideal detection time will occur when the train head is at the end of the switch. The ideal matched filter has 34 coefficients, providing a slightly delayed peak with respect the approximated rectangular version with 30 coefficients. The output voltage has been scaled with a constant gain block in order to obtain the processing scale factor of the real track detector, implemented in the system FPGA and Single Board Computer.

The noise spectrum and noise power evolution along the simulated sensor processing chain has been estimated when the yaw rate signal is absent (H0). Using a large signal power averaging in order to obtain statistically significant values, the standard deviation results are very close to theoretical predictions. This confirms the correct design of the multirate processing approach. In the 5 km/h velocity simulated case, the noise standard deviation obtained at the output of the ideal matched filter is σ_y_ = 91.02 mV and the signal peaks at 6.212 V, resulting in a simulated signal-to-noise ratio *S/N*_ideal_ = 36.68 dB, which is very close the theoretical value *Ŝ* / *N* = 36.87 dB obtained in (17). In the case of the rectangular matched filter the noise typical deviation is 95.17 mV with a peak signal of 6.137 V and a *S/N*_rect_ = 36.19 dB showing a degradation of about 0.5 dB with respect to the ideal filter

The simulation has also been carried out for the maximum velocity of 50 km/h, with results, shown in Figure 14, very similar to those obtained at 5 km/h taking into account the amplitude 1/10 scale-factor of the gyro signal and the 10 scale-factor of the elapsed time. The amplitude-scale differences between the 5 and 50 km/h runs are removed, as expected, by the accumulator delivering a velocity-independent spatial sampling of the turnout signal. The signal-to-noise ratio is benefitted by the larger yaw rates obtained by the gyro in the 50 km/h case. The theoretical *Ŝ* / *N* value can be easily calculated from the expected impact of train velocity on the matched filter signal-to-noise ratio analyzed in Section 5, resulting in *Ŝ* / *N* (50 km/h) = 10· *Ŝ* / *N* (5 km/h) = 46.87 dB. As in the baseline 5 km/h case, the simulated values are very close the theoretical reference: *S/N*_ideal_ = 46.5 dB and *S/N*_rect_ = 46.0 dB.

## 7. Experimental Evaluation of the Detector Performance

**Figure 16.**
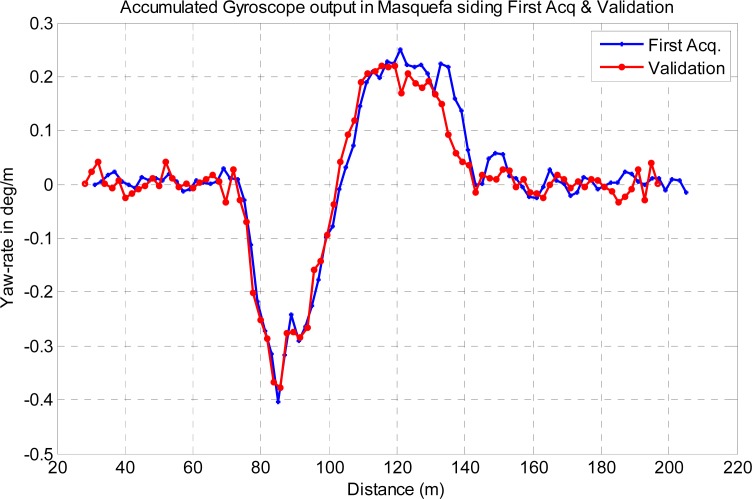
Spatial yaw rate signal obtained in Masquefa siding in the first acquisition and validation campaigns.

The proposed detector performance has been experimentally assessed using the gyroscope acquired data in the two available acquisition campaigns. Figure 16 shows the spatial yaw rate signals expressed in degrees per meter (deg/m), delivered by the distance sampler accumulator of Figure 10, in the Masquefa station test siding. Two plots are shown, corresponding respectively to the first gyroscope signal acquisition, based on a low accuracy average velocity measurement of the train tachometer, and to the validation campaign with the gyroscope acquisition and accumulator processing embedded in the BLOCKSAT^®^ system. The typical train deceleration when arriving to the station has not been taken into account in the first acquisition campaign, because of the non-availability of the constant distance pulses, but has been accurately compensated in the validation campaign by the accumulator; this fact explains the spatial yaw rate discrepancies around the distance of 135 meters.

In absence of turnout (H0) the output of the accumulator has been assumed to be sensor noise only. The potential impact of train vibration on the gyroscope noise budget has been estimated from the gyroscope acceleration-rejection data [10] and the typical acceleration experimentally measured on trains [24], resulting in interfering signals in the order of 2% of the noise power, confirming the sensor internal-noise assumption.

**Figure 17.**
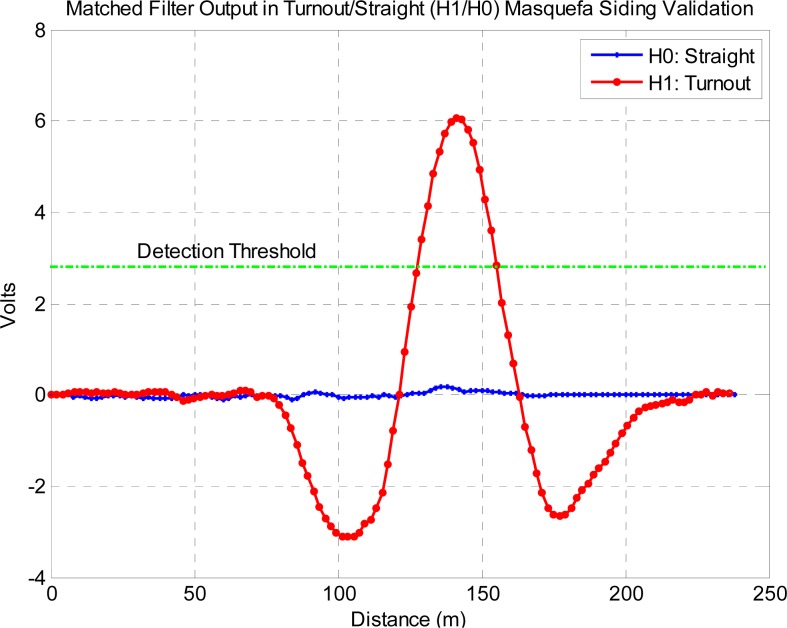
Output of the matched filter in the H0 and H1 trajectories obtained in the Validation 2011 campaign. The horizontal line shows the threshold proposed decision level.

The siding detection has been tested on the experimental data acquired in the validation campaign in December 2011 for both H0 (main) and H1 (siding) tracks using the simplified rectangular weight version of the matched filter. Figure 17 shows the matched filter output for the H0 and H1 cases.

The matched filter output in both cases H0 and H1 shows a very similar noise standard deviation, which is 0.052 deg/m in the H0 case. The signal peak in the H1 case is 6.028 volts, a value that includes the gyroscope yaw-rate sensitivity, accumulator scale factor and the unit weights and number of branches of the matched filter. This peak value is proportional to the yaw rate signal energy [8] and is in good agreement with both theoretical and simulated results shown in Figures 13 and 14. The experimental signal-to-noise ratio is S/N = 41.3 dB, in the order of 2 dB below the theoretical optimum value at the train velocity of 28.8 km/h. The 2 dB difference can be attributed in part to the non-ideal matched filter, that has been characterized by simulation and shown to introduce around 0.5 dB loss, and in another part to excessive gyroscope bandwidth.

In the analysis the turn (D1) or no-turn (D0) decision has been assumed to occur close to the matched filter output peak. Since the train position will not be perfectly known in practice, some position tolerance should be accounted for by arming and disarming the threshold detector around the expected peak position. In order to maintain the intended probabilities of error, the length of the interval of detection should be established according to the position error statistics. In the BLOCKSAT^®^ case the specified maximum position error derived from duplicated Doppler radar odometers and differential GPS receivers is 10 m. This allows dimensioning the detection interval in the order of the correlation length of the matched filter output, which has been estimated as the inverse of the noise equivalent output signal bandwidth, or, in terms of travelled distance, *L_c_* ≈ 24 m.

If larger decision intervals are required, the evaluation of false alarms must consider the effective number of independent decisions *N_d_* involved, which can be estimated from the ratio between the spatial interval of decision *L_d_* and the correlation length *L_c_* where the matched filter output is assumed to be correlated:

(23) $$N_{d}≅\frac{L_{d}}{L_{c}}$$

After *N_d_* independent decisions the cumulative probability of false alarm *P_cfa_*[9] can be obtained as a particular case of the binomial distribution where one or more positive detections results in a false alarm:

(24) $$P_{cfa}=1-{\left(1-P_{fa}\right)}^{Nd}≅N_{d}\cdot P_{fa}\;\;\;\;\;\;\;\;\;\;\mathrm{if}\;\;\;\;\;\;\;\;\;\;\;\;\;P_{\mathrm{fa}}\ll 1$$

In any case, with the proposed processing chain the detection errors are extremely improbable, since taking a threshold equal to the half peak value as was shown in Section 5, *P_fa_* = *P_m_* ≪ 10^−9^.

## 8. Conclusions

This paper presents an optimized processing chain and detection providing reliable decision on track occupancy in railway sidings based on yaw rate signals provided by MEMS gyroscopes. The binary detection problem has been stated for typical turnout geometries. It has been shown that a simple yaw rate threshold detector offer acceptable performance at moderate to high velocities, but does not provide the required probabilities of detection error at slow train maneuvering velocities.

An improved detector based on a two-stage multi-rate matched filter has been proposed, showing a remarkable improvement over simple threshold detectors. In this case the required error probabilities are achieved with a large signal-to-noise safety margin, even at the slowest operational velocities (5 km/h). The implementation aspects of the detector have been optimized for real-time operation in a recently developed BLOCKSAT^®^ blocking system that includes an accurate navigation subsystem. For optimum operation the detector filter must be matched to the gyroscope yaw rate signal expected in the turnout, dependent on the rail road geometry and train velocity, which cannot be assumed constant. For this reason the real-time velocity obtained from Doppler radar odometer combined with differential GPS is used to convert the acquired time-domain signal into spatial-domain yaw rate data. A digital accumulator implemented in the BLOCKSAT^®^ system FPGA provides the signal time-to-space scaling, amplitude compensation and adaptive low-pass filter, minimizing both the processed noise power and the spatial sampling. In this way, the spatial correlation of the matched filter can be obtained with a small amount of operations that can be further minimized for a constant weight approximate implementation.

The detector has been studied theoretically, by computer simulations and experimentally on a representative railway test case. The experimental results have confirmed the theoretical and simulated analysis, resulting in a reliable detection. In the proposed system, all the sensors and processing modules are duplicated for higher reliability and integrity. The gyroscopes and associated electronics can be periodically tested and calibrated on train stops and on straight and curved segments of the rail track. The gyroscope derived yaw rate patterns reveal small details of the track geometries and could be exploited for additional applications, like assessing track deformation and degradation of switch mechanisms. Present research is addressed to extend the proposed track detector to more general cases including sidings implemented on curved main tracks.
